# Pre-Conception Dyslipidemia Is Associated with Development of Preeclampsia and Gestational Diabetes Mellitus

**DOI:** 10.1371/journal.pone.0139164

**Published:** 2015-10-09

**Authors:** Yael Baumfeld, Lena Novack, Arnon Wiznitzer, Eyal Sheiner, Yakov Henkin, Michael Sherf, Victor Novack

**Affiliations:** 1 Soroka Clinical Research Center, Soroka University Medical Center, Be’er-Sheva, Israel; 2 Department of Obstetrics and Gynecology, Soroka University Medical Center, Be’er-Sheva, Israel; 3 Department of Public Health, Faculty of Health Sciences, Ben-Gurion University of the Negev, Be’er-Sheva, Israel; 4 Department of Cardiology, Soroka University Medical Center, Be’er-Sheva, Israel; 5 Medical Administration, Clalit Health Services, Tel-Aviv, Israel; INIA, SPAIN

## Abstract

**Introduction:**

The association between glucose intolerance, elevated blood pressure and abnormal lipid levels is well established and comprises the basis of metabolic syndrome pathophysiology. We hypothesize that abnormal preconception lipid levels are associated with the increased risk of severe pregnancy complications such as preeclampsia and gestational diabetes mellitus.

**Methods:**

We included all singleton deliveries (n = 27,721) of women without known cardiovascular morbidity and preeclampsia and gestational diabetes mellitus during previous pregnancies. Association between preconception low high density lipoprotein cholesterol (HDLc level≤50 mg/dL), high triglycerides (level≥150 mg/dL) and the primary outcome (composite of gestational diabetes mellitus/or preeclampsia) was assessed using Generalized Estimation Equations.

**Results:**

Primary outcome of preeclampsia and/or gestational diabetes was observed in a total of 3,243 subjects (11.7%). Elevated triglycerides and low HDLc were independently associated with the primary outcome: with odds ratio (OR) of 1.61 (95% CI 1.29–2.01) and OR = 1.33 (95% CI 1.09–1.63), respectively, after adjusting for maternal age, weight, blood pressure, repeated abortions, fertility treatments and fasting glucose. There was an interaction between the effects of HDLc≤50 mg/dL and triglycerides≥150 mg/dL with an OR of 2.69 (95% CI 1.73–4.19).

**Conclusions:**

Our analysis showed an increased rate of preeclampsia and/or gestational diabetes in women with low HDLc and high triglycerides values prior to conception. In view of the severity of these pregnancy complications, we believe this finding warrants a routine screening for the abnormal lipid profile among women of a child-bearing age.

## Background

Adult metabolic syndrome is defined by a combination of elevated blood glucose, central obesity, elevated blood pressure, high triglycerides and low high density lipoproteins cholesterol (HDLc). Insulin resistance, an underlying pathology of the metabolic syndrome, also is a known risk factor for development of pregnancy complications such as hypertensive disorders of pregnancy (HDP) comprising of gestational hypertension, preeclampsia/eclampsia and preeclampsia superimposed on chronic hypertension and gestational diabetes mellitus (GDM). Indicating possible common pathophysiology, women with gestational diabetes mellitus are also at increased risk for preeclampsia [1, 2]. Both of these pregnancy-related disorders are associated with development of future chronic diseases, i.e. type 2 diabetes and hypertension. [3]

Normal pregnancy is associated with a physiological increase in total cholesterol, triglycerides and lipoproteins [4, 5]. However, abnormally elevated triglycerides (the additional component of the metabolic syndrome), total cholesterol and low density lipoprotein cholesterol (LDLc) measured in pregnancy were all found to be associated with preeclampsia [1, 6, 7, 8]. High gestational triglyceride levels alone were shown to have an adverse impact on the composite outcome of preeclampsia and /or gestational diabetes mellitus [6].

Pregestational lipid levels (triglycerides, LDLc, total cholesterol) were found to be associated with premature birth and low birth weight [9]. However elevated triglycerides levels and low levels of HDLc, both components of metabolic syndrome were not studied in relation to future pregnancy complications. In the current study we aimed to investigate the independent impact of an elevated lipid profile prior to gestation on the composite outcome of preeclampsia and gestational diabetes mellitus.

## Methods

### Study population

In this investigation we used prospectively-collected data on all deliveries between January 2005 and December 2011 in the Soroka University Medical Center (SUMC). SUMC is a 1000 bed tertiary teaching hospital and the only provider for inpatient care for a population of 700,000 residing in Southern Israel. During the study period average annual number of deliveries managed at SUMC was around 12,500.

In the study population we included women who had one of the lipids tests (analyzed in the single laboratory) in a time frame of 12 months prior to the conception of a singleton pregnancy. Women with ischemic heart disease, stroke, peripheral vascular disease, known diabetes mellitus diagnosed prior to conception, dyslipidemia or hypertension were excluded from the analysis. This database derived from the computerized retrospective database of the medical Center. Data collected in the database included information on maternal comorbidities, perinatal assessment, maternal and fetal complications. Fasting glucose levels during gestation were assessed as well. In a case of a multiple glucose tests the maximal value was chosen.

### Lipids levels measurement

The timing of lipid testing was related to the nearest estimated conception date. The cut-off points were defined as HDLc level≤50 mg/dL and fasting triglycerides level≥150 mg/dL following National Cholesterol Education Program and American Heart Association guidelines [10]. In case of multiple tests during the 12 months prior to the conception, the tests with highest triglycerides and lowest HDLc levels were chosen.

### Definitions

Composite diagnosis of preeclampsia and/or gestational diabetes mellitus was the primary composite outcome in the study. The outcome diagnoses were assessed as recorded by a treating physician according to standard definitions used in our institution. Preeclampsia was defined as blood pressure of 140/90 mmHg after 20 weeks gestation and proteinuria of ≥300 mg of protein in 24 hours.

*Gestational diabetes mellitus (GDM)* was diagnosed based on results of a universal screening, i.e. a GCT of over 200 mg/dL or an abnormal OGTT test (two pathological measurements out of the 4-fasting measurements—1 hour, 2 hours or 3 hours after ingestion of 100 mg glucose).

*Preterm delivery* was defined as delivery prior to 37 weeks of gestation, small for gestational age (SGA) as birth weight below 10^th^ percentile for gestational age and large to gestational age (LGA) weight above 90^th^ percentile.

*Repeated spontaneous abortion* was defined as two or more spontaneous abortions.

### Data analysis

Each delivery was treated as a separate observation in the univariate analysis. The data on continuous variables with normal distribution were presented as mean ± SD, and compared between study groups using Student t-test. Continuous variables not normally distributed and ordinal variables were presented as median with inter-quartile range (IQ range) and statistical analysis was done using Mann-Whitney. Categorical data were shown in counts and percentages and the differences were assessed by Chi-Square, Fisher Exact test was used when appropriate.

Association between low HDLc, high triglycerides and the primary composite outcome was assessed by a multivariable logistic regression model using Generalized Estimation Equation (GEE) method, which accounted for clusters of multiple deliveries by the same women. Covariance matrix was conservatively defined as "unstructured". Variable selection in multivariable modeling was based on clinical and statistical significance. The following variables were included into the models: maternal age, preconception weight, blood pressure and preconception total cholesterol and glucose levels (in increments of 10 mg/dL), gravidity, fertility treatment, history of the recurrent abortions and. HDLc and triglycerides were included into the models stratified into four groups as following: We reported final parsimonious models.

Logistic regression models were used to create local regression (LOESS) curves for the likelihood of primary outcome (composite of GDM and preeclampsia) as a function of HDLc and triglycerides, adjusted for the age, gravidity, abortions, fertility treatments and weight.

Two-sided p-value of <0.05 was considered significant.

### Ethics

The study was approved by the "Soroka University Medical Center" Ethics Committee. All clinical investigation was conducted according to the principles expressed in the Declaration of Helsinki. Informed consent was not required having that the data was collected retrospectively maintaining subject confidentiality. Patient records was anonymized and de-identified prior to analysis.

## Results

A total of 89,051 deliveries (50,845 women) took place in Soroka University Medical Center between January 2005 and December 2011. Lipid assessment prior to conception was performed in 27,721 cases (31.1%, 15,222 women). Women without a lipid test, and therefore not included in the study population, were slightly older (29.5±5.8 vs. 28.0±5.7 years, p<0.001), had higher gravidity (4.1±3.0 vs. 3.5±2.6, p<0.001) and higher rate of repeated spontaneous abortions (5.9% vs. 4.6%, p<0.001). GDM and preeclampsia rates were lower in the non-tested population (3.7% vs. 4.5% and 5.3% vs. 4.3%, respectively, p<0.001 for both). The rest of the maternal and neonatal characteristics were similar between the groups.

### Population characteristics

Primary composite outcome of preeclampsia and/or gestational diabetes was observed in a total of 3,243 deliveries (3,131 women). Table 1 compares maternal and neonatal characteristics women with and without an outcome. Women who developed the composite outcome were significantly older (32.4 vs. 30.1, p<0.001), had higher gravidity (4.6 vs. 4.0, p<0.001) and parity (3.8 vs. 3.4, p<0.001). They also had a higher rate of repeated spontaneous abortions and fertility treatments (9.4% vs 6.4% and 10.3% vs. 4.9% respectively, p<0.001 for both).

**Table 1:** Maternal characteristics, peripartum complications, neonatal outcomes, and preconception lipid levels in the study population.

Variables	Preeclampsia or GDM	No preeclampsia or GDM	P value
	N = 3243	N = 24421	
Age, years. mean±SD	32.4±6.1	30.1±5.7	<0.001
History of repeated spontaneous abortions n, (%)	306 (9.4)	1556 (6.4)	<0.001
History of infertility treatments n, (%)	335 (10.3)	1188 (4.9)	<0.001
Gravidity, Median (IQ range)	3 (2–7)	3 (2–5)	<0.001
Gravidity 1 (%)	930 (13.4)	6032 (86.6)	<0.001
Gravidity2-5 (%)	1478 (9.6)	13985 (90.4)	<0.001
Gravidity6+ (%)	834 (16.0)	4383 (84.0)	<0.001
Parity, Median (IQ range)	3 (1–6)	3 (2–5)	0.01
Parity1 (%)	712 (12.6)	4953 (87.4)	<0.001
Parity2-5 (%)	1496 (9.9)	12583 (90.1)	<0.001
Parity6+ (%)	1035 (15.0)	5871 (85.0)	<0.001
Gestational age at birth, weeks, median (IQ range)	38.0 (37.0–39.7)	39.0 (38.0–40.0)	<0.001
Systolic blood pressure, mmHg, mean±SD	125.9±17.6	115.3±13.4	<0.001
Diastolic blood pressure, mmHg, mean±SD	78.78±11.4	72.01±15.7	<0.001
Maternal Weight before pregnancy (kg), Mean±SD	80.9±17.8	70.1±16.2	<0.001
Pre-gestation laboratory results, Mean±SD			
Glucose, mg/dL	111.03±33.85	101.90.28.89	<0.001
LDLc, mg/dL	108.1±29.6	101.8±29.9	<0.001
HDLc, mg/dL	52.9±13.3	55.0±13.1	<0.001
Total cholesterol, mg/dL	191.9±41.5	180.4±41.5	<0.001
Triglycerides, mg/dL	147.5±88.9	110.9±66.3	<0.001
Hemoglobin, g/dL	12.1±0.9	11.9±0.9	<0.001
Creatinine, mg/dL	0.5±0.2	0.54±0.1	0.050
Newborn Weight (grams), Mean±SD	3081.2±753.8	3145.6±579.9	<0.001

### HDLc and triglycerides levels

Women with preeclampsia and/or gestational diabetes had higher preconception glucose (123 vs. 106 mg/dL), LDLc (108 vs. 102 mg/dL), total cholesterol (192 vs. 180 mg/dL) and triglycerides (147 vs. 111 mg/dL) and a lower HDLc (53 vs. 55 mg/dL).

We compared between patients with triglycerides levels≥150 mg/dL and patients with triglycerides levels<150 mg/dL (Table 2). Women in the group with higher triglycerides levels were heavier (77 vs. 70 kg), had higher LDLc (117 vs. 99 mg/dL, p<0.001), higher total cholesterol levels (217 vs.171 mg/dL, p<0.001) and higher systolic and diastolic blood pressure (121/75 vs. 116/72, p<0.001 for both). HDLc levels were similar between the two groups (54 vs. 55 mg/dL, p = 0.03). Women with higher triglycerides had higher rates of various gestational complications, e.g. mal presentation (10 vs. 8%, p<0.001), premature delivery (14 vs. 11%, p<0.001) and large for gestational age newborns (10 vs. 7%, p<0.001).

**Table 2:** Comparison of the study population by pre-gestation triglycerides below and above 150 mg/dL.

Variables	Triglycerides ≥150 mg/dL	Triglycerides <150 mg/dL	P value
	N = 5667	N = 19294	
Age, years mean ±SD	31.7±5.9	30.1±5.7	<0.001
History of repeated spontaneous abortions, n (%)	452 (8.0)	1231 (6.4)	<0.001
History of Infertility treatments, n (%)	353 (6.2)	1086 (5.6)	0.090
Gravidity, Median (IQ range)	4 (2–7)	3 (2–5)	<0.001
Gravidity 1 (%)	1137 (18.2)	5116 (81.8)	<0.001
Gravidity 2–5 (%)	3088 (22.2)	10840 (77.8)	<0.001
Gravidity 6+ (%)	1439 (30.2)	3321 (69.8)	<0.001
Parity, Median (IQ range)	3 (2–6)	3 (1–4)	<0.001
Parity 1 (%)	885 (17.4)	4208 (82.6)	<0.001
Parity2-5 (%)	2960 (21.8)	10608 (78.2)	<0.001
Parity 6+ (%)	1821 (29.0)	4465 (71.0)	<0.001
Gestational age, weeks median (IQ range)	39.0 (37.7–40.0)	39.0 (38.0–40.0)	<0.001
Systolic blood pressure, mmHg, mean±SD	120.7±16.5	115.6±13.7	<0.001
Diastolic blood pressure, mmHg, mean±SD	75.0±13.9	72.3±15.3	<0.001
Maternal Weight before pregnancy (kg), Mean±SD	76.9±17.3	70.0±16.34	<0.001
Newborn Weight (grams), Mean±SD	3154.7±647.5	3137.3±585.6	0.070
Pre-gestation laboratory results, Mean±SD			
Glucose, mg/dL	106.50±31.51	102.13±29.12	<0.001
LDLc, mg/dL	117.1±35.6	98.9±27.1	<0.001
HDLc, mg/dL	54.3±15.0	54.81±12.61	0.030
Total cholesterol, mg/dL	216.8±45.8	171.2±33.5	<0.001
Triglycerides, mg/dL	216.4±74.7	85.6±29.8	<0.001
Hemoglobin, g/dL	11.9±1.0	12.0±0.9	<0.001
Creatinine, mg/dL	0.5±0.2	0.6±0.1	<0.001
Pregnancy complication (%)			
Mal presentation	592 (10.5)	1561 (8.1)	<0.001
Premature delivery	786 (13.9)	2140 (11.1)	<0.001
Premature rapture of membranes	598 (10.6)	2401 (12.5)	<0.001
Large for gestational age	572 (10.2)	1301 (6.8)	<0.001
Small for gestational age	213 (3.8)	714 (3.7)	0.850

We further compared patients stratified by HDLc levels prior to conception using a cut-off of 50 mg/dL (Table 3). Age was similar between the groups. Low HDLc group was characterized by the higher incidence of the repeated spontaneous abortions. The main differences included lower LDLc (98 vs. 105 mg/dL, p<0.001) and total cholesterol (165 vs. 190 mg/dL, p<0.001) along with greater weight (75 vs. 70 kg, p<0.001) and triglyceride level (119 vs. 108 mg/dL, p<0.001) in the group of women with low HDLc levels. This group had higher rates of mal presentation (10 vs. 8%, p<0.001).

**Table 3:** Comparison of the study population by pre-gestation HDLc levels below and above 50 mg/dL.

Variables	HDLc≤50 mg/dL	HDLc≥50 mg/dL	p-value
	N = 8088	N = 13,154	
Age, years mean±SD	30.7±6.0	30.7±5.6	0.700
History of repeated spontaneous abortions, n (%)	632 (7.8)	785 (6.0)	<0.001
History of Infertility treatments, n (%)	488 (6.0)	781 (6.0)	0.780
Gestational age at delivery, median (IQ range)	39.0 (38.0–40.0)	39.0 (38.0–40.0)	0.590
Systolic blood pressure, mmHg, mean±SD	118.1±14.8	116.3±14.3	<0.001
Diastolic blood pressure, mmHg, mean±SD	73.6±15.5	72.6±15.8	<0.001
Maternal weight, kg, mean±SD	74.7±17.6	70.6±16.4	<0.001
Pre-gestation Laboratory results, mean±SD			
Glucose, mg/dL	103.64±30.07	103.03	0.16
LDLc, mg/dL	98.0±27.2	105.4±31.2	<0.001
HDLc, mg/dL	42.3±5.3	62.3±10.5	<0.001
Total cholesterol, mg/dL	164.6±33.5	189.9±40.5	<0.001
Triglycerides, mg/dL	119.4±72.3	108.5±64.8	<0.001
Hemoglobin, g/dL	12.0±0.9	12.0±0.9	0.540
Creatinine, mg/dL	0.5±0.1	0.6±0.1	<0.001
Pregnancy complications (%)			
Mal presentation	775 (9.6)	1026 (7.8)	<0.001
Premature delivery	1014 (12.5)	1469 (11.2)	0.010
Premature rapture of membranes	929 (11.5)	1632 (12.4)	0.050
Large for gestational age	633 (7.9)	968 (7.4)	0.210
Small for gestational age	305 (3.8)	477 (3.7)	0.580
Newborn weight in grams, mean±SD	3147.8±634.0	3145.5±579.7	0.790

### Primary outcome

**Fig 1:**
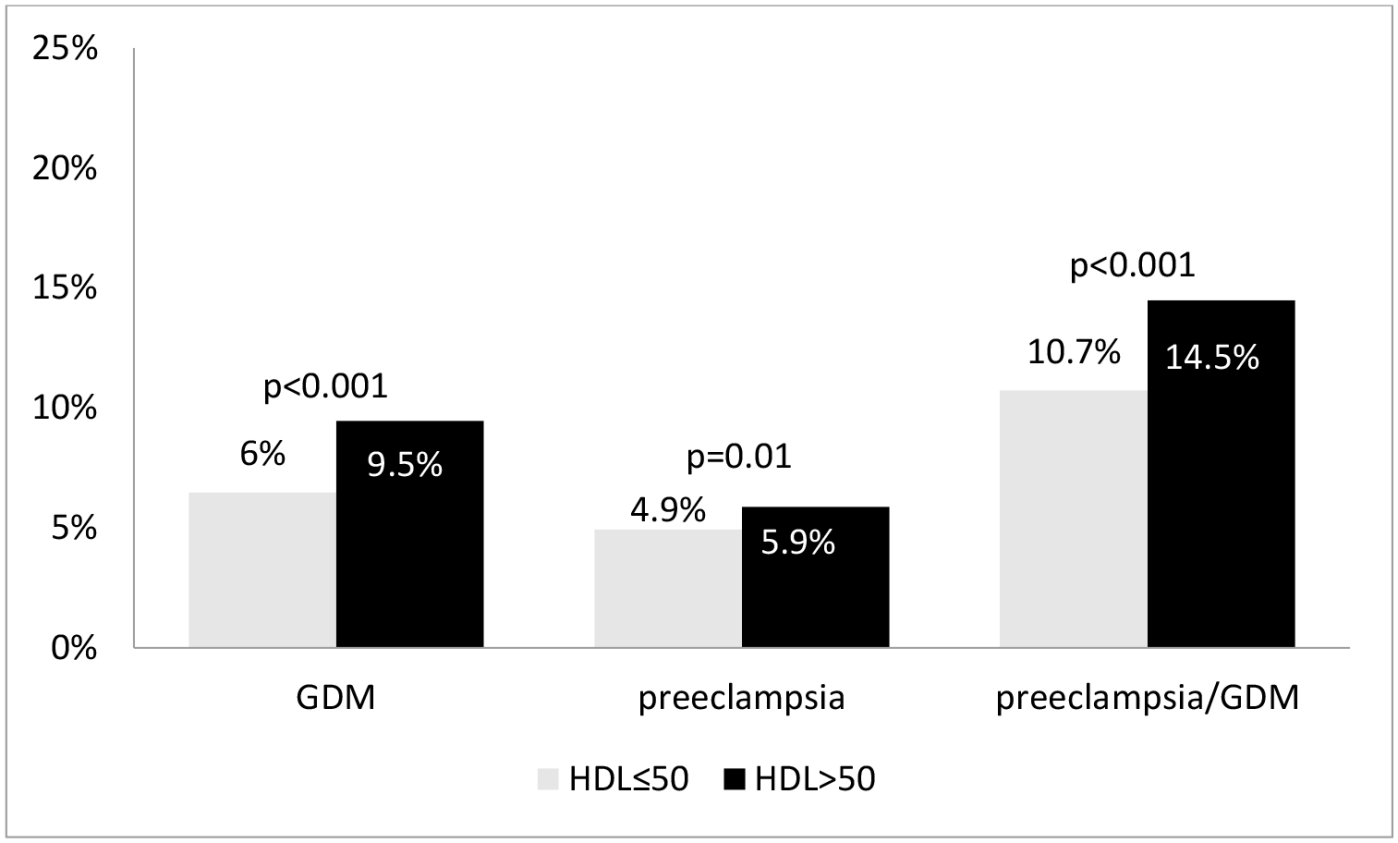
Outcome rates in patients with triglyceride level above and below 150 mg/dL.

**Fig 2:**
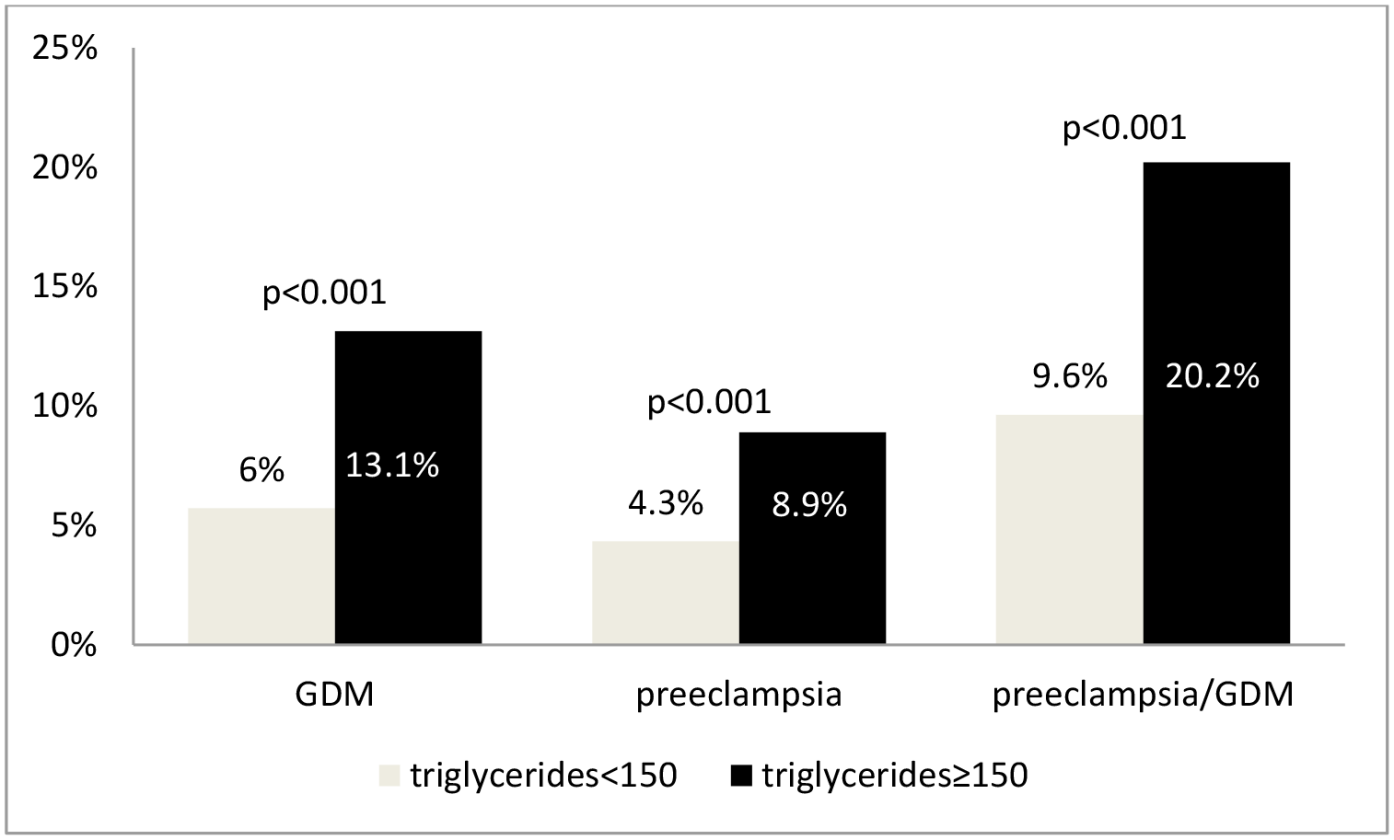
Outcome rates in patients with HDL level above and below 50 mg/dL.

Women with low HDLc levels had the rate of the primary outcome of preeclampsia and/or GDM of 14.5%% vs. 10.7%% in the group with higher HDLc (Fig 1, p<0.001). Similarly, women with high triglycerides had the rate of the primary outcome of 20.2%% vs. 9.6%% in the group with lower triglycerides (Fig 2, p<0.001). In a subgroup of women with both elevated triglycerides and low HDLc (n = 1928) the rate of the primary outcome was 24.7% as compared to 9.1% in the group with low triglycerides and high HDLc (p<0.001).

Elevated triglycerides (≥150 mg/dL) and low HDLc (≤50 mg/dL) were independently associated with the primary outcome: with odds ratio (OR) of 1.61 (95% CI 1.29–2.01) and OR = 1.33 (95% CI 1.09–1.63), respectively, after adjusting to established risk factors for GDM/preeclampsia, including maternal age, weight, blood pressure, habitual abortions, fertility treatments and fasting glucose. There was an interaction between the effects of HDLc≤50 mg/dL and triglycerides≥150 mg/dL with an OR of 2.69 (95% CI 1.73–4.19) (Table 4).

**Table 4:** General estimating equation (GEE) for the prediction of the composite outcome (preeclampsia/GDM).

Variables	OR	95% CI	P value
Age, years	1.08	1.06–1.10	<0.001
Weight, kg	1.02	1.01–1.02	<0.001
Systolic blood pressure, mmHg	1.04	1.03–1.05	<0.001
Fertility treatments	1.60	1.12–2.28	0.01
History of repeated abortions	1.80	1.32–2.44	<0.001
Parity	0.91	0.88–0.94	<0.001
Glucose by 10 mg/dL	1.05	1.02–1.09	0.01
Compared to triglycerides<150 and HDLc>50 mg/dL			
Triglycerides ≥150 mg/dL	1.61	1.29–2.01	<0.001
HDLc, mg/dL ≤50	1.33	1.09–1.63	0.01
TGL≥150 and HDLc≤50 ng/dL	2.32	1.80–2.99	<0.001

Multivariate analysis of the composite outcome was repeated stratified by the glucose cut-off point used for the definition of diabetes mellitus: >125 mg/dL and ≤125 mg/dL. The adjusted risks were similar in both groups: OR = 1.57 for triglycerides≥150 mg/dL, OR = 1.30 for HDLc≤50 mg/dL in groups of women with glucose>125 mg/dL (n = 5322, 19.2%) and OR = 1.61, OR = 1.35 for women with glucose levels≤125 mg/dL (n = 21226, 76.6%).

Furthermore, elevated triglycerides levels were shown to be independently associated with the primary outcome with an OR of 2.38 and with each of the components of the outcome with an OR = 2.16 and OR = 2.50 for preeclampsia and GDM, respectively (p<0.001 for all). Similarly, low HDLc levels were independently associated with the primary outcome with an OR of 1.41 and with each of the components of the outcome with an OR = 1.20 and OR = 1.51 for preeclampsia and GDM, respectively (p<0.001 for all). LDLc levels were not found to be associated with preeclampsia and /or gestational diabetes.

**Fig 3:**
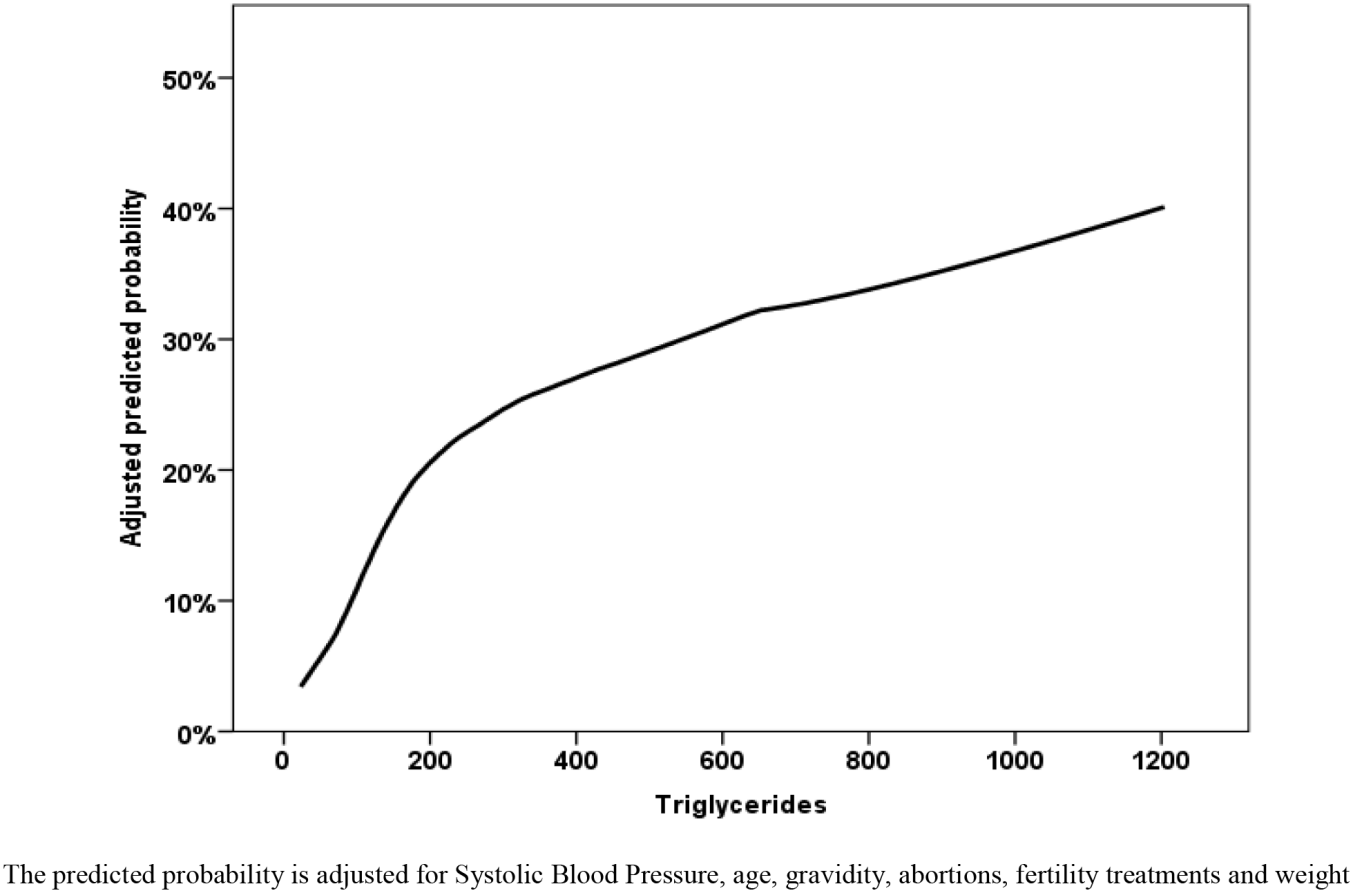
Locally weighted scatterplot smoothing (LOESS) non parametric regression for the prediction of the GDM or preeclampsia composite outcome.

**Fig 4:**
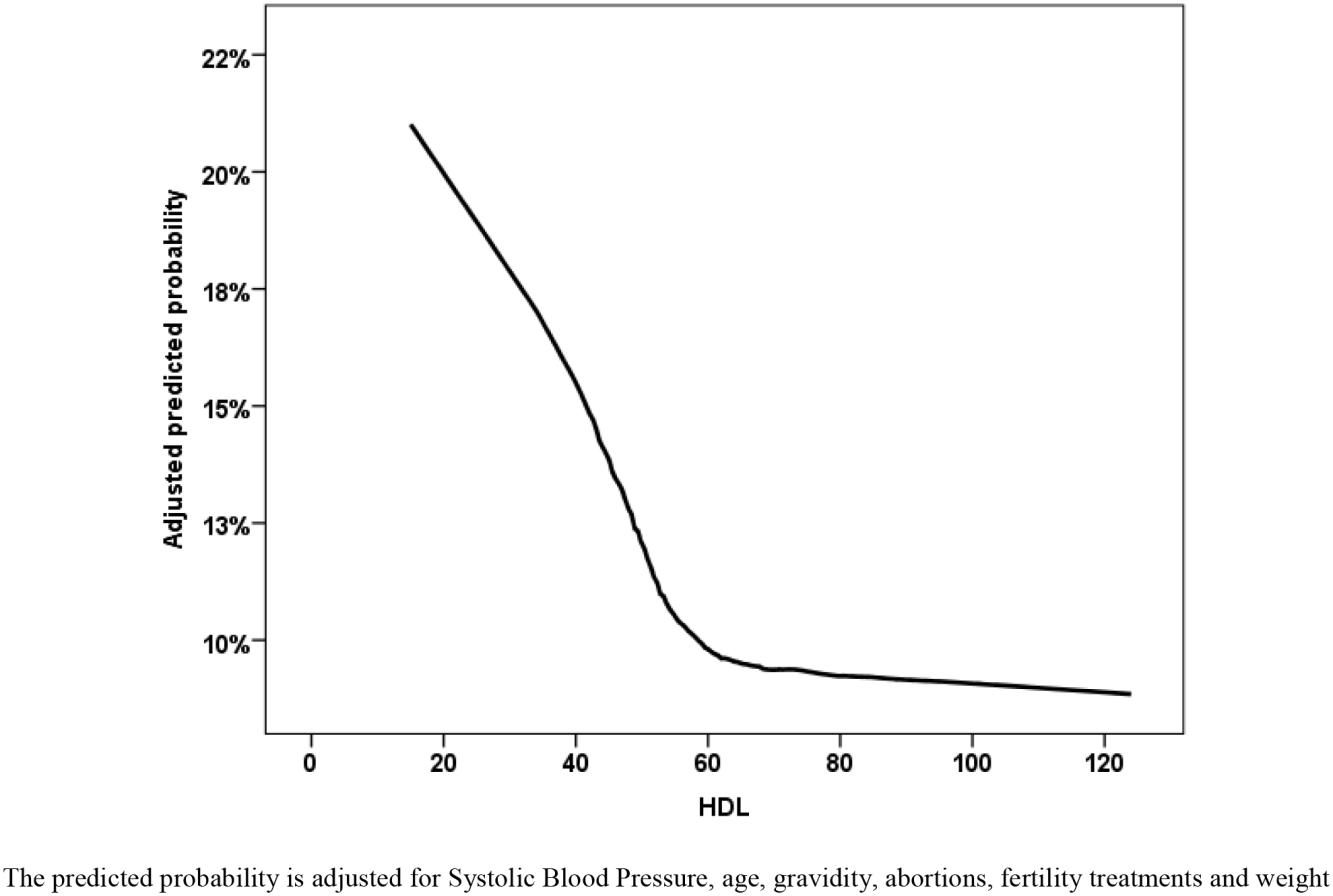
Locally weighted scatterplot smoothing (LOESS) non parametric regression for the prediction of the GDM or preeclampsia composite.

To assess the dose dependency between HDLc, triglycerides and outcome we used LOESS approach with an adjustment to systolic blood pressure, age, gravidity, history of abortions, fertility treatments and weight and glucose levels before pregnancy (Figs 3 and 4). We found an almost linear association between adjusted triglycerides levels and the outcome, while the effect of low adjusted HDLc levels is plateauing above the level of 60 mg/dL.

## Discussion

We found that low preconception levels of HDLc and high levels of triglycerides are independently associated with an increased risk for preeclampsia and/or gestational diabetes mellitus, while the highest rates of this composite outcome were observed in a group with both high triglycerides and low HDLc.

In this study we studied the primary hypothesis that an association between the components of the metabolic syndrome prior to conception and possible presentations of the gestational metabolic syndrome. Specifically, we explored the association between abnormal lipid levels and the composite outcome of gestational diabetes and hypertensive disorders of pregnancy. Based on our dose-response adjusted analysis we were able to establish an almost linear positive association between pre-gestation triglycerides levels and the outcome and a negative linear effect HDLc levels for values below 60 mg/dL. The observed associations were adjusted for the various maternal characteristics inclusive of fasting glucose, weight, blood pressure, age and parity. The associations remained significant with as similar effect size in group of women with possibly undiagnosed diabetes mellitus defined by glucose levels above 125mg/dL.

Different aspects of the cardiovascular risk factors effect on pregnancy outcomes have been studied. Elevated blood pressure was found to be a risk factor for preeclampsia [6, 11]. Body fat percentage was found to be associated with gestational diabetes and hypertension risks [11] and BMI with lipid level changes during pregnancy [12, 13, 14, 15]. Harville et al. showed that pre-pregnancy components of metabolic syndrome including high BMI, triglycerides, waist circumference and low HDLc are associated with an increased risk of gestational diabetes [16]. In our previous study we have explored the effect of gestational changes in lipid levels on the pregnancy outcomes [17], however so far no cut-off points for the preconception lipid levels were defined. Our current analysis further reinforces the association of both preeclampsia and gestational diabetes with abnormal lipids levels. Elevated triglyceride levels and/or low HDLc cholesterol levels, both of which are part of the metabolic syndrome, usually precede the onset of hyperglycemia in individuals prone to develop diabetes [17, 18]. Thus, we hypothesize that the risk of pregnancy complications associated with elevated triglycerides and low HDLc may be the manifestation of the metabolic syndrome of pregnancy. I.e. subtle signs of metabolic syndrome prior to a conception can be translated into a detrimental health effect during pregnancy, a natural state of insulin resistance, in susceptible women.

What do our findings mean for women contemplating conception and their physicians? We believe that these findings can lead to an addition of lipids screening to the pre-pregnancy assessment. Following identification of women with abnormal lipid levels, increased vigilance for early signs of pre-eclampsia and GDM might be considered. This includes close blood pressure monitoring and a glucose tolerance test [19]. Life style change for women found to have abnormal metabolic profile can be recommended. These interventions can be undertaken both before and during the gestation.

Dietary changes have been shown to improve maternal metabolic profile and specifically lipids homeostasis [20–23]. Exercise is a common lifestyle change advocated for dyslipidemia and its effect during pregnancy has been explored in a number of studies, showing a decrease in LDL levels [24], trend toward lowering free fatty acids [25] and reduction in fasting triglycerides levels [26].

Supplementation of omega 3 fatty acids can be considered as another therapeutic option. Studies have shown a decrease in triglyceride levels in the general population [27]. Furthermore multiple studies have explored their effect on different pregnancy outcomes thus providing reassurance regarding the safety profile.

The study strength and limitations are inherent to the retrospective design. The large population based contemporary cohort with a negligible loss to follow up increases results generalisability, however, this methodology did not allow for a control over the type of the data accumulated in the database. For instance, no measures of height were available, and therefore the analysis used weight for adjustment, rather than a more relevant measure of a body mass index. The study population was limited to those who had a lipid level test, exposing the study analysis and its conclusions to a selection bias. However, the comparison between women with and without lipids testing in health related characteristics did not reveal major differences. Furthermore, the rates of the outcomes between tested and non-tested women were similar, reassuring that the extent of the potential selection bias is somewhat minimal.

We believe that the findings in this study have great significance for further screening and treatment of major obstetric pathologies i.e. gestational diabetes mellitus and preeclampsia. We also believe further studies are needed to explore the cut point for triglyceride levels, treatment options including lifestyle changes, and omega 3 supplements including the timing, dosage and lipid level treatment goals.

To conclude, there was an increased rate of preeclampsia and gestational diabetes in women with low HDLc and high triglycerides values measured prior to conception. In view of high severity of the two pregnancy complications—the finding may warrant routine screening for the abnormal lipid profile, especially among women planning pregnancy and having an elevated risk of gestational diabetes and/or hypertensive disease of pregnancy.

## References

[1] Kaaja R, Laivuori H, Laakso M, Tikkanen MJ, Ylikorkala O: Evidence of a state of increased insulin resistance in preeclampsia. Metabolism 1999; 48(7): 892–6. 10421232 10.1016/s0026-0495(99)90225-1

[2] Emonts P, Seaksan S, Seidel L, et al: Prediction of Maternal Predisposition to Preeclampsia. Hypertension in Pregnancy 2008; 27(3): 237–245. 10.1080/10641950802000901 18696352

[3] Nerenberg K, Daskalopoulou SS, Dasgupta K. Gestational diabetes and hypertensive disorders of pregnancy as vascular risk signals: an overview and grading of the evidence. Can J Cardiol. 2014 7;30(7):765–73. 10.1016/j.cjca.2013.12.030 24726053

[4] Bartels Ä, Egan N, Broadhurst DI, Khashan AS, Joyce C, Stapleton M, O'Mullane J, O'Donoghue K. Maternal serum cholesterol levels are elevated from the 1st trimester of pregnancy: a cross-sectional study. J Obstet Gynaecol. 2012 11;32(8):747–52. 10.3109/01443615.2012.714017 23075347

[5] Maseki M, Nishigaki I, Hagihara M, Tomoda Y, Yagi K. Lipid peroxide levels and lipids content of serum lipoprotein fractions of pregnant subjects with or without pre-eclampsia. Clin Chim Acta. 1981 9 10;115(2):155–61 7285362 10.1016/0009-8981(81)90071-1

[6] Magnussen EB, Vatten LJ, Lund-Nilsen TI, Salvesen KA, Davey Smith G, Romundstad PR: Prepregnancy cardiovascular risk factors as predictors of pre-eclampsia: population based cohort study. BMJ 2007; 335(7627): 978 17975256 10.1136/bmj.39366.416817.BE PMC2072028

[7] Ray JG, Diamond P, Singh G, Bell CM: Brief overview of maternal triglycerides as a risk factor for pre-eclampsia. BJOG 2006; 113(4): 379–86. 16553649 10.1111/j.1471-0528.2006.00889.x

[8] Bukan N, Kandemir O, Nas T, Gulbahar O, Unal A, Cayci B.J. Maternal cardiac risks in pre-eclamptic patients. Matern Fetal Neonatal Med. 2012 7;25(7):912–4 10.3109/14767058.2011.600363 21827368

[9] Catov JM, Bodnar LM, Kip KE, et al Early pregnancy lipid concentrations and spontaneous preterm birth. Am J Obstet Gynecol 2007;197:610.e1–610.e7. 18060950 10.1016/j.ajog.2007.04.024

[10] ATP III Final Report PDF: Third Report of the National Cholesterol Education Program (NCEP) Expert Panel on Detection, Evaluation, and Treatment of High Blood Cholesterol in Adults (Adult Treatment Panel III) Final Report Circulation. 2002;106:25 3143. 12485966

[11] Zhao YN, Li Q, Li YC. Effects of body mass index and body fat percentage on gestational complications and outcomes. J Obstet Gynaecol Res. 2014 3;40(3):705–10. 24738116 10.1111/jog.12240

[12] Baksu B, Baksu A, Davas I, Akyol A, Gülbaba G. Lipoprotein levels in women with pre-eclampsia and in normotensive pregnant women. J Obstet Gynaecol Res. 2005 6;31(3):277–82. 15916667 10.1111/j.1447-0756.2005.00276.x

[13] Hedderson MM, Darbinian JA, Sridhar SB, Quesenberry CP. Prepregnancy cardiometabolic and inflammatory risk factors and subsequent risk of hypertensive disorders of pregnancy. Am J Obstet Gynecol. 2012 7;207(1):68 e1–9. 10.1016/j.ajog.2012.05.017 22727352 PMC4161150

[14] Enquobahrie DA, Williams MA, Butler CL, Frederick IO, Miller RS, Luthy DAAm J Maternal plasma lipid concentrations in early pregnancy and risk of preeclampsia Hypertens. 2004 7;17(7):574–81. 10.1016/j.amjhyper.2004.03.666 15233976

[15] Vahratian A, Misra VK, Trudeau S, Misra DP. Prepregnancy body mass index and gestational age-dependent changes in lipid levels during pregnancy. Obstet Gynecol. 2010 7;116(1):107–13. 20567175 10.1097/AOG.0b013e3181e45d23

[16] Harville EW, Juonala M, Viikari JS, Raitakari OT. Preconception metabolic indicators predict gestational diabetes and offspring birthweight. Gynecol Endocrinol. 2014 11;30(11):840–4. 10.3109/09513590.2014.937336 25007009

[17] Wiznitzer A, Mayer A, Novack V, Sheiner E, Gilutz H, Malhotra A et al Lipids during gestation with preeclampsia and gestational diabetes mellitus: a population based study. AJOG 2009. 10.1016/j.ajog.2009.05.032 PMC5483324 19631920

[18] Taskinen MR. Diabetic dyslipidaemia: from basic research to clinical practice. Diabetologia. 2003; 46: 733–749. 12774165 10.1007/s00125-003-1111-y

[19] Ginsberg HN, Zhang YL, Hernandez-Ono A. Metabolic syndrome: focus on dyslipidemia. Obesity (Silver Spring). 2006; 14: 41S–49S. 16642962 10.1038/oby.2006.281

[20] Hoppu U, Isolauri E, Koskinen P, Laitinen K Nutrition. Maternal dietary counseling reduces total and LDL cholesterol postpartum 2014 2;30(2):159–64. 10.1016/j.nut.2013.07.009 24176529

[21] Barrett HL, Nitert ND, McIntyre HD Callaway LK Normalizing metabolism in diabetic pregnancy: is it time to target lipids? Diabetes Care. 2014 5;37(5):1484–93. 10.2337/dc13-1934 24757231

[22] Aucott L, Gray D, Rothnie H, Thapa M, Waweru C. Effects of lifestyle interventions and long-term weight loss on lipid outcomes—a systematic review. Obes Rev. 2011 5;12(5):e412–25. Epub 2011 Mar 4. 10.1111/j.1467-789X.2010.00819.x 21371252

[23] Khoury J, Henriksen T, Christophersen B, Tonstad S. Effect of a cholesterol-lowering diet on maternal, cord, and neonatal lipids, and pregnancy outcome: a randomized clinical trial. Am J Obstet Gynecol. 2005 10;193(4):1292–301. 16202717 10.1016/j.ajog.2005.05.016

[24] Loprinzi PD, Fitzgerald EM, Woekel E, Cardinal BJ. Association of physical activity and sedentary behavior with biological markers among U.S. pregnant women. J Womens Health (Larchmt). 2013 11;22(11):953–8. 23968237 10.1089/jwh.2013.4394 PMC3820143

[25] Hopkins SA, Baldi JC, Cutfield WS, McCowan L, Hofman PL. Effects of exercise training on maternal hormonal changes in pregnancy. Clin Endocrinol (Oxf) 2011;74:495–500 21198740 10.1111/j.1365-2265.2010.03964.x

[26] Hollingsworth DR, Moore TR. Postprandial walking exercise in pregnant insulin-dependent (type I) diabetic women: reduction of plasma lipid levels but absence of a significant effect on glycemic control. Am J Obstet Gynecol. 1987 12;157(6):1359–63. 3425644 10.1016/s0002-9378(87)80224-7

[27] Kastelein JJ, Maki KC, Susekov A, Ezhov M, Nordestgaard BG, Machielse BN et al Omega-3 free fatty acids for the treatment of severe hypertriglyceridemia: the EpanoVa fOr Lowering Very high triglyceridEs (EVOLVE) trial. J Clin Lipidol. 2014 Jan-Feb;8(1):94–106. 10.1016/j.jacl.2013.10.003 24528690

