# Correction: Pre-Conception Dyslipidemia Is Associated with Development of Preeclampsia and Gestational Diabetes Mellitus

**DOI:** 10.1371/journal.pone.0142462

**Published:** 2015-11-04

**Authors:** Yael Baumfeld, Lena Novack, Arnon Wiznitzer, Eyal Sheiner, Yakov Henkin, Michael Sherf, Victor Novack

The Funding section is incorrect. The correct Funding information is: This study was supported by the research grant from Marie Skłodowska-Curie Fund, European Commission (268184).
